# Bacterial global regulators DksA/ppGpp increase fidelity of transcription

**DOI:** 10.1093/nar/gkv003

**Published:** 2015-01-20

**Authors:** Mohammad Roghanian, Nikolay Zenkin, Yulia Yuzenkova

**Affiliations:** Centre for Bacterial Cell Biology, Institute for Cell and Molecular Biosciences, Newcastle University, Baddiley-Clark Building, Richardson Road, Newcastle upon Tyne NE2 4AX, UK

## Abstract

Collisions between paused transcription elongation complexes and replication forks inevitably happen, which may lead to collapse of replication fork and could be detrimental to cells. Bacterial transcription factor DksA and its cofactor alarmone ppGpp were proposed to contribute to prevention of such collisions, although the mechanism of this activity remains elusive. Here we show that DksA/ppGpp do not destabilise transcription elongation complexes or inhibit their backtracking, as was proposed earlier. Instead, we show, both *in vitro* and *in vivo*, that DksA/ppGpp increase fidelity of transcription elongation by slowing down misincorporation events. As misincorporation events cause temporary pauses, contribution to fidelity suggests the mechanism by which DksA/ppGpp contribute to prevention of collisions of transcription elongation complexes with replication forks. DksA is only the second known accessory factor, after transcription factor Gre, that increases fidelity of RNA synthesis in bacteria.

## INTRODUCTION

Multi-subunit DNA-dependent RNA polymerase (RNAP) is highly processive and can continue RNA synthesis for thousands of nucleotides without dissociation from the template DNA or the RNA product. Transcription, however, can frequently be interrupted by pauses of various nature (1,2). Pausing can be caused by signals in the nucleic acids of the elongation complex (EC) that impede incorporation of nucleoside monophosphate (NMP) by altering properties of the active centre (elemental pauses) (2,3) or slow down forward translocation by RNAP (translocation pauses) (4,5). Either of these pauses can also lead to backtracking of RNAP, a phenomenon when the 3′ end of RNA disengages from the active centre and RNAP shifts backward along the template. Backtracking can also occur upon misincorporation events, when the non-cognate NMP at the 3′ end of RNA forces RNAP into 1 base pair backtracked state (6,7). This also may stimulate further backtracking. While elemental and translocation pauses are usually relatively short-living, backtracking, if not resolved, frequently represents a dead-end event (8).

Recently, we proposed that backtracked complexes may cause queuing of the trailing RNAPs, which results in ‘traffic jams’, which, in turn, strongly impede gene expression (9). Furthermore, in bacteria, there is no temporal separation of the transcription and replication machineries that share the same DNA template (10). With the rate of replication being approximately 20-fold greater than the rate of transcription, collisions are inevitable and thought to occur frequently (11–14). Paused, backtracked and, particularly, queuing transcription elongation complexes are potent obstacles to replication forks. Backtracked elongation complexes were recently shown to impede the replication fork and result in chromosomal double-strand breaks (15). Failure to deal with such conflicts has been reported to result in genome instability, including chromosomal rearrangements and deletions (16–18).

Translating ribosomes and trailing RNAPs (when multiple RNAPs are transcribing the same gene) have been suggested to suppress backtracking and promote the forward movement of a stalled RNAP (15,19–21). More recent study, however, suggested that this may not be enough, and trailing RNAPs form ‘traffic jams’, at least in some bacterial species, which must be even more potent obstacles for replication forks (9). To efficiently deal with conflicts between the replication and transcription machineries bacteria employ a number of elongation factors (13–14,22–23). Anti-backtracking factor Mfd (a double-stranded DNA translocase) binds to stalled elongation complexes and the upstream DNA (24). Translocation of Mfd along the DNA has been shown to revive the backtracked RNAP to resume transcription (24) or to displace stalled complex from DNA (13,25,26). Gre factors impose a highly effective hydrolytic activity to the active centre of RNAP, which resolves misincorporated/backtracked complexes and restores the 3′ end of RNA in the active centre allowing further extension (27). Recently, we proposed that resolution of paused (via misincorporation or backtracking) complexes is the major, if not the only, function of Gre in the cell, at least in some bacterial species (9).

A ∼17 kD structural homologue of Gre, transcription factor DksA, was reported to reduce the need for replication fork repair after collisions with transcribing RNAPs (13–14,22,28). DksA is best known to act synergistically with global regulator of transcription initiation, alarmone ppGpp, by potentiating the effect of ppGpp on transcription initiation (29), though the exact mechanism of this synergy remains unclear. Recent data showed DksA was enriched at not only the promoter region but across the entire transcription unit (30). DksA is thought to act through RNAP secondary channel. However, during elongation DksA, unlike Gre factors, does not impose cleavage or, apparently, any other activity to the RNAP's active centre that could potentially resolve backtracked complexes. It was suggested that DksA prevents collisions with replication fork possibly by destabilising elongation complexes (13,14) or by inhibiting RNAP backward movement (30). However, another report suggested that, at least *in vitro*, DksA does not bind to backtracked or active elongation complexes (31). Another study suggested that DksA has an inhibiting effects on RNA elongation (32). ppGpp was also linked to prevention of interference of transcription and replication, as it was shown to reduce accumulation of arrays of stalled elongation complexes (13). Whether ppGpp may directly affect elongation complexes is not known. Taken together, the mechanism by which DksA with or without ppGpp participates in resolving conflicts between the replication and transcription remains unclear.

Here we show that, in contrast to previous models, DksA neither destabilises elongation complexes, nor inhibits their backward movement. Instead, DksA, with aid from ppGpp, increases the fidelity of RNA synthesis, and thus possibly prevents formation of misincorporated elongation complexes that interfere with replication.

## MATERIALS AND METHODS

### Proteins and reagents

Wild type *Escherichia coli* RNAP and DksA were purified by standard procedures as previously described (29,33). Oligonucleotides were from Integrated DNA Technologies (IDT). ppGpp was from TriLink BioTechnologies. ΔDksA and its parental wild type (BW25113) strains were from Keio collection (34). Plasmid pUV12 coding for β-galactosidase gene without premature stop codon was developed by Vogel and Jensen (35). The stop codon was introduced at codon +17 using standard mutagenesis (plasmid pUV12stop).

### Transcription assays

All transcription experiments were done at 37°C in transcription buffer containing 20 mM Tris-HCl pH 7, 40 mM KCl, 10 mM MgCl_2_, unless otherwise specified. EC11 was obtained on a templates containing T7A1 promoter as previously described (8), except that the complexes were immobilized on streptavidin agarose beads (Fluka) through biotin of the 5′-end of the non-template DNA strand. EC11 was labeled in the body with α[^32^P]GMP, or was obtained unlabeled and ‘walked’ as described (36) to positions +26 and +31 followed by labeling at the 3′end through incorporation of α[^32^P]UMP to obtain EC27 and EC32, respectively. EC27 (labeled at 0°C to prevent backtracking) was left to backtrack at 37°C for the times given in the figures and/or the figure legends. For elongation complex stability experiments EC11 and EC32 were left for times indicated in transcription buffer containing either 500 mM KCl or 100 μg/mL heparin before separation of supernatant and bound fractions. Transcription from rrnB P1 promoter was performed in the same conditions as for T7A1, except for using priming dinucleotide CpA and labeling with α[^32^P]CMP. Artificial elongation complexes were assembled and immobilized exactly as described (37). RNA was labeled at the 3′-end by incorporation of α[^32^P]GMP after complex assembly as described (38). For all experiments, DksA and ppGpp were added before reactions for 1 min to 5 μM or 0.1 mM, respectively. For exonucleolytic cleavage reaction in Supplementary Figure S1B ppGpp was added to 1 mM.

After incubation for times specified in figures or the figure legends, reactions were stopped with formamide containing buffer. Products of all reactions were resolved by denaturing PAGE (8 M Urea), revealed by PhosphorImaging (GE Healthcare) and analyzed using ImageQuant software (GE Healthcare). Kinetic data were fitted to a single exponential equation using non-linear regression in SigmaPlot (38).

The β-galactosidase assays were performed exactly as described (39), with the exception that the expression from T7A1 promoter containing two *lac* operators was induced with IPTG for 45 min. Production of β-galactosidase from pUV12stop (with premature stop codon) was on average 9.6 ± 0.7 times higher in ΔDksA strain than in wild-type strain. Differential expression of β-galactosidase from pUV12 (without premature stop codon) in ΔDksA and wild-type strains was similar after 10 and 45 min (even though absolute values between 10 and 45 min differed ∼10-fold) indicating that no saturation in the amount of β-galactosidase in the cell has been reached. Production of β-galactosidase from pUV12 was on average 2.3 ± 0.4 times higher in ΔDksA strain than in wild-type strain. To account for that the absolute values of β-galactosidase assay with pUV12stop were divided by 2.3 for ΔDksA strain. All experiments were repeated at least three times.

## RESULTS

### DksA/ppGpp do not have significant effects on transcription elongation

As mentioned in the Introduction, the data and proposals on the effects of DksA on elongation appear to be contradictory. The aim of our study was to explore the possible mechanisms that allow DksA to prevent collisions of transcription ECs with replication forks.

**Figure 1.**
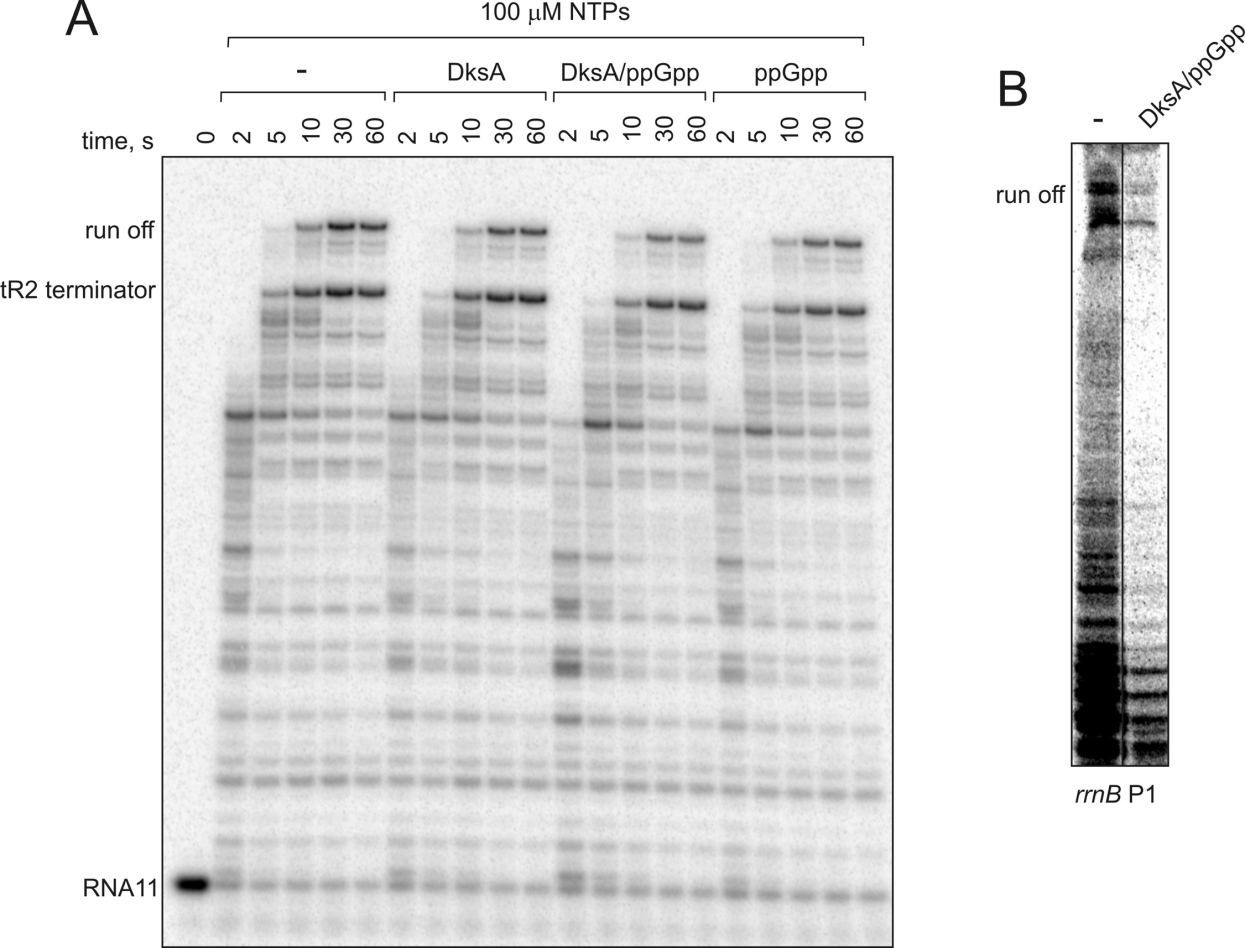
DksA and ppGpp have a minor inhibitory effect of transcription elongation. (**A**) Chase (100 μM NTPs) of stalled elongation complex EC11, obtained from T7A1 promoter and containing 11 nucleotide long RNA (RNA11), in the presence or absence of 5 μM DksA and/or 100 μM ppGpp (here and after). (**B**) Multi-round transcription in 300 μM NTPs on the polymerase chain reaction fragment containing *rrnB* P1 promoter. Here and after, black vertical line separates lanes that originate from one gel and were brought together.

We tested the effects of DksA and ppGpp on transcription elongation on a well studies template containing T7A1 promoter (8,36,40). Transcription by *E. coli* RNAP was initiated in conditions of incomplete set of nucleoside triphosphates (NTPs) to form a stable elongation complex containing 11 nucleotide-long RNA (EC11). Transcription then was resumed by addition of 100 μM NTPs in the absence or presence of 5 μM DksA and/or 100 μM ppGpp (concentrations close to those found in the cell (41)). As seen from Figure 1A, DksA alone or with ppGpp (DksA/ppGpp) had a weak inhibitory effect on elongation. The result is in good agreement with the earlier work (32). No stimulation of transcription or overcoming of pauses was observed, which somewhat contradicts the proposal that DksA may suppress pausing (30).

We tested whether our purified DksA was functional. DksA in conjunction with ppGpp decreases the activities of some promoters, and in particular those coding for components of the protein synthesis machinery (29,42–43). A well-characterized promoter that is strongly inhibited by DksA and ppGpp is *rrnB* P1 (29,44). To check the DksA activity, we analyzed transcription on a DNA fragment containing the *rrnB* P1 promoter. As seen from Figure 1B, upon addition of 5 μM DksA and 100 μM ppGpp we observed strong inhibition of transcription consistent with earlier observations (29,44). This confirmed that our preparation of DksA was active. Taken together the above results suggest that, at least *in vitro*, DksA cannot suppress pausing or robustly stimulate transcription.

### DksA/ppGpp do not destabilise elongation complexes

**Figure 2.**
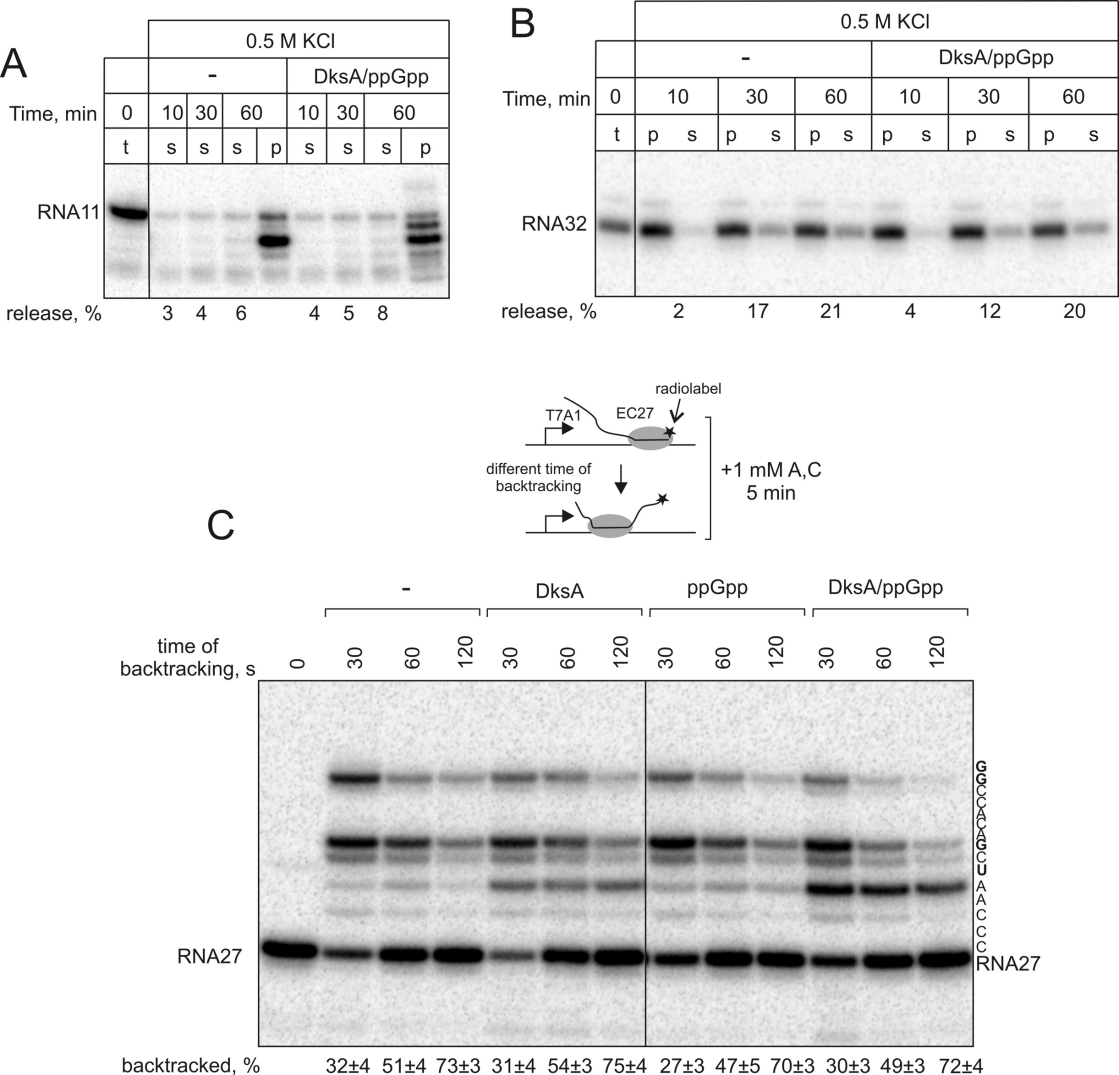
DksA and ppGpp do not affect the stability or backtracking of elongation complexes. (**A** and **B**) EC11 and EC32 obtained from T7A1 promoter were immobilized on streptavidin beads, and supernatant (s) and/or pellet (p) fractions were analyzed after various times of incubations in transcription buffer containing 0.5 M KCl (for low salt conditions see Supplementary Figure S1A). Percentage of RNA released into the supernatant fraction is shown below the gels. Different cleavage patterns in case of RNA11 were due to ppGpp induced exonucleolytic cleavage (see Supplementary Figure S1B and text). (**C**) The scheme of the experiment is shown next to the gel. Backtracking-prone EC27 obtained from T7A1 promoter. RNA27 was labeled at the 3′ end by incorporation of α[^32^P]-UMP. Complexes were allowed to backtrack for various times in the presence or the absence of DksA and ppGpp. Backtracking of EC27 was monitored by loss of ability to extend RNA27 after various times of incubation. Percentage of backtracked complexes is shown below the gel (averages and standard deviations from four independent experiments, ±SD). Note strong read-through via misincorporation at the positions shown in bold within transcribed sequence to the right of the gel.

As DksA/ppGpp had no apparent stimulatory effects on transcription elongation, we tested whether DksA/ppGpp could destabilise elongation complexes, as was previously proposed (13,14). To investigate this suggestion, the stalled EC11 and EC32 formed on T7A1 template were immobilized on streptavidin beads via a biotin tag on the non-template strand. This allowed us to analyse possible destabilisation of the elongation complexes by detecting the release of the radiolabeled RNA into the reaction solution. We found that destabilisation by DksA/ppGpp was not strong enough to result in release of RNA in low salt conditions (Supplementary Figure S1A). To spot even small differences in stability of elongation complexes we used transcription buffer with high monovalent salt concentration (0.5 M KCl), a common method for measuring stability of elongation complexes (45). As seen from Figure 2A and B, the presence of DksA and ppGpp made no difference on the release of RNA into the reaction solution in either EC11 or EC32. Note that EC11 has almost fully undergone intrinsic hydrolysis during the time of incubation, indicating that it is prone to backtracking. Therefore, the absence of release of RNA from EC11 suggests that DksA/ppGpp also cannot destabilise backtracked complexes, such as misincorporated ones. Altogether, the results indicate that DksA/ppGpp do not affect the stability of the elongation complexes, at least *in vitro*.

We noticed the pattern of RNA cleavage in the presence of DksA/ppGpp was different in the presence and absence of DksA/ppGpp (Figure 2A, Supplementary Figure S1A). Investigation of this phenomenon revealed that ppGpp was responsible for stimulating the exonucleolytic cleavage of RNA (Supplementary Figure S1B). ppGpp changed cleavage patterns differently in different elongation complexes (compare EC11 in low and high salt conditions, and EC32), likely due to different translocational or possible conformational states of the active centre in these complexes. Non-complementary NTPs were shown to catalyse exonucleolytic cleavage by stabilising the second catalytic Mg^2+^ ion in the active centre (46). It is possible that ppGpp can also stabilise Mg^2+^ ion by binding in the active centre as was proposed earlier (47). We however cannot exclude the possibility that ppGpp allosterically changes translocation and/or catalytic properties of the active centre by binding at ω subunit of RNAP (48–50).

### DksA and ppGpp do not prevent backtracking of elongation complex

DksA was suggested to alleviate detrimental effects of collisions between the replication and the transcription machineries by inhibiting backtracking of RNAP (14,30). Therefore, next, we analyzed possible effects of DksA/ppGpp on backward movement of RNAP.

A well-studied backtracking-prone elongation complex, EC27, was formed on T7A1 template and labeled at the 3′ end of the RNA. Last step of EC27 formation was done at 0°C to preclude backtracking. Backtracking can then be stimulated by raising temperature to 37°C. Falling of EC27 into backtracking can be monitored by its ability to elongate RNA after different times of incubation at 37°C. Analysis of the ability of EC27 to extend RNA would report on even short backtracking, although EC27 eventually backtracks by 16 base pairs (8). EC27 were allowed to backtrack for the indicated times and then were supplied with 1 mM ATP and CTP to allow the non-backtracked complexes to elongate. As seen from Figure 2C, DksA, with or without ppGpp, had no effect on backtracking of the complexes. The results thus suggest that prevention of collisions of replication machinery with the elongation complexes by DksA is unlikely to be due to inhibition of backtracking.

### DksA and ppGpp decrease misincorporation by RNAP

Addition of ATP and CTP in the above experiment (Figure 2C) should allow EC27 to elongate only by 5 nucleotides, to position +32 (see sequence to the right of the gel). As seen from Figure 2C, however, RNAP reads-through to positions +34 (misincorporating at +33dAMP) and position +35 (misincorporation at +35dCMP) and even elongates further. The read-through, however, was much less in the presence of DksA/ppGpp in the reaction.

**Figure 3.**
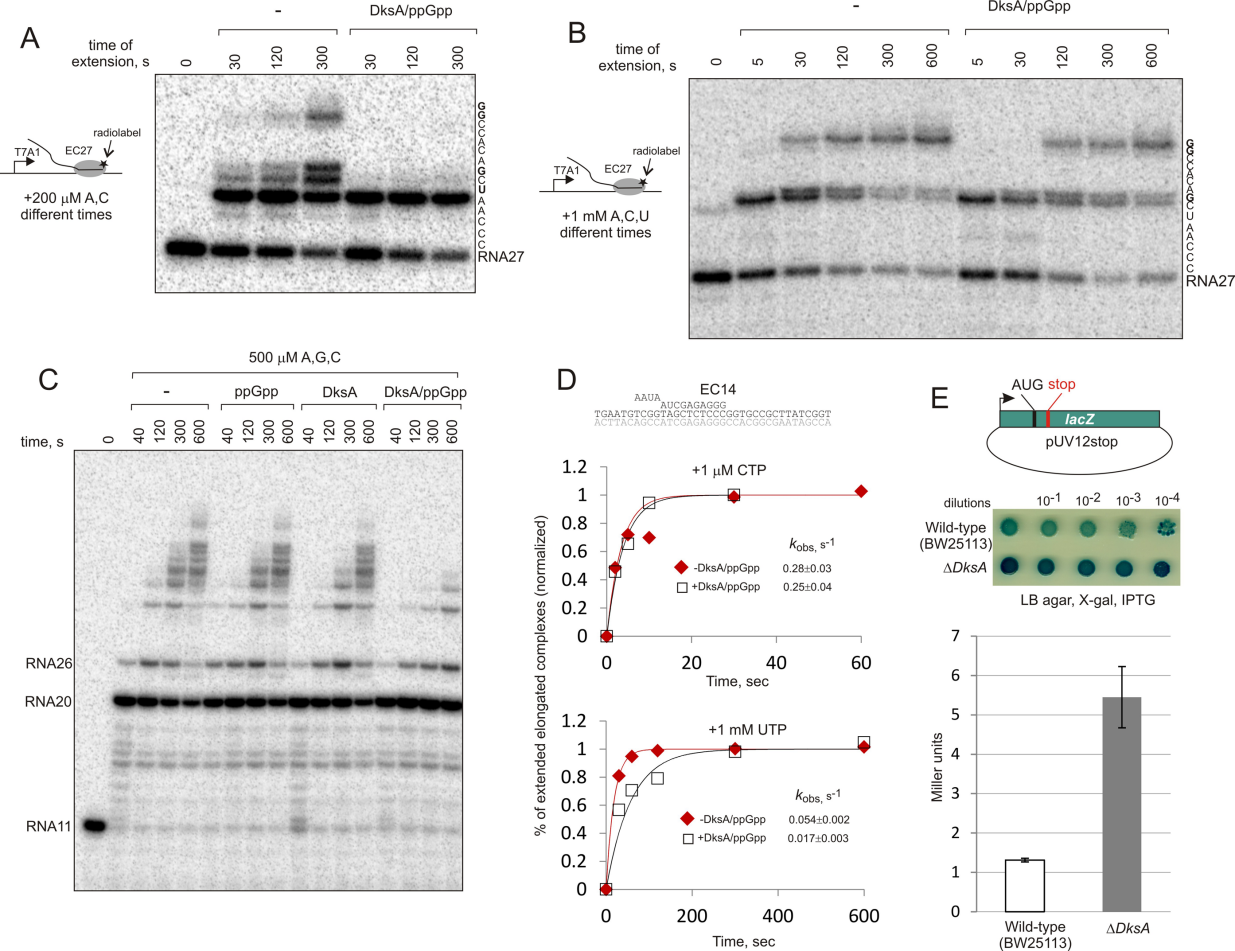
DksA/ppGpp slows down misincorporation by RNAP. (**A** and **B**) Extension of EC27 in the presence of ATP and CTP (A) or ATP, CTP and UTP (B). Note that DksA/ppGpp decrease the read-through at positions shown in bold within transcribed sequences to the right of the gels. (**C**) Chase of EC11 in the presence of 500 μM ATP, GTP and CTP (compare to chase in all NTPs in Figure 1A). (**D**) Kinetics of incorporation (1 μM CTP) and misincorporation (1 mM UTP) in the assembled elongation complex (shown above the plots), in the presence or absence of DksA/ppGpp. Observed rate constants (*k*_obs_) were obtained by a fit of the averages from two independent experiments into single-exponential equation (numbers that follow the ± sign are standard errors). The plots were normalized to the predicted maximum which was taken as 1. (**E**) Plasmid pUV12stop carrying *lacZ* gene with a premature stop codon at position 17 (scheme at the top) was transformed into wild-type and ΔDksA *E. coli* strains (Keio collection (34)). Production of active β-galactosidase through transcription misincorporation (51) was visualized on the agar plate containing X-gal by spotting serial dilutions of wild-type and ΔDksA cultures (the dark spots, which we do not know the reason for, were observed at higher dilutions of many different strains with normal and decreased fidelities that we tested in this assay (not shown)), and quantified in liquid β-galactosidase assay. The absolute values for ΔDksA strain were divided by 2.3 to account for differential regulation of expression of the construct in wild-type and ΔDksA strains as measured by levels of expression of *lacZ* gene from pUV12 (without premature stop codon) in these strains (see Materials and Methods for details). Error bars are standard deviation from three independent experiments.

To test this phenomenon in more details we analyzed kinetics of elongation of RNA27, without allowing EC27 to backtrack prior to the addition of ATP and CTP (Figure 3A). As seen, DksA/ppGpp clearly slowed down the misincorporation at +33dAMP. When EC27 was supplied with ATP, CTP and UTP (which must allow extension to position +34) a strong read-through was observed at +35dCMP. However, presence of DksA/ppGpp slowed down misincorporation at +35dCMP, and thus decreased the read-through.

Similar result was observed during elongation of EC11 in the presence of incomplete set of NTPs; DksA/ppGpp strongly decreased misincorporation and read-throughs (Figure 3C), while, as we showed above, had little effect on transcription in the presence of all four NTPs (Figure 1A). The results suggest that DksA/ppGpp increase fidelity of transcription possibly by slowing down misincorporation by RNAP.

To directly test how DksA/ppGpp may affect the fidelity of transcription, we analyzed rates of misincorporation of a single nucleotide with or without DksA/ppGpp using assembled elongation complex (scheme in Figure 3D). As seen from Figure 3D, DksA/ppGpp indeed slowed down misincorporation of 1 mM NTP, while having no effect on incorporation of even 1 μM correct NTP. The result confirmed that DksA/ppGpp may increase fidelity of transcription. Misincorporated complexes cause transcription pauses (6,7) and may lead to further backtracking. Therefore, though DksA cannot directly inhibit backtracking, the anti-misincorporation activity of DksA/ppGpp may explain their positive effect on transcription elongation *in vivo* and prevention of collisions of elongation complexes with replication forks (see Discussion).

To test if DksA can contribute to the fidelity of transcription *in vivo*, we transformed wild type (WT) and a deleted for DksA (ΔDksA) *E. coli* strains with the plasmid (pUV12stop), which contained IPTG-inducible *lacZ* gene with a premature stop codon at position +17 (scheme in Figure 3E). Expression of the active β-galactosidase from such construct reports on misincorporation at this stop codon during transcription, while translational read-through of stop codon appears to be insignificant comparing to transcription errors (51). Though inducible T7A1 promoter in this construct is thought not to be directly affected by DksA/ppGpp, to account for their possible effects on expression, we first measured β-galactosidase production from β-galactosidase gene without premature stop codon (plasmid pUV12) in WT and ΔDksA strains (see Materials and Methods for details). β-galactosidase production from pUV12 in ΔDksA strain was 2.3 ± 0.4 fold higher than in WT strain. Therefore, the expression values obtained from pUVstop in ΔDksA strain were divided by 2.3 before plotting (Figure 3E). As seen from Figure 3E, deletion of DksA resulted in significant increase of β-galactosidase production from pUV12stop indicative of suppression of the stop codon, which suggests the higher transcription error rate in ΔDksA strain. This result supports the above *in vitro* results that DksA may contribute to transcription fidelity.

## DISCUSSION

The above *in vitro* data suggest that the ability of DksA to prevent interference between transcription elongation and replication cannot be directly explained by either prevention of backtracking, or overcoming of transcription pauses, or destabilisation of elongation complexes.

The principal finding of this work is that DksA/ppGpp increase fidelity of RNA synthesis by slowing down incorporation of erroneous nucleotides by RNAP. Misincorporation causes backtracking by one base pair and, thus, a temporary pause of transcription. This can also stimulate further backtracking, which may lead to formation of arrested complex. Backtracked complexes are proposed to form potent obstacles for a replication fork. Therefore, while not being able to inhibit backtracking *per se*, DksA may prevent formation of misincorporated backtracked complexes and thus may explain the anti-collision properties of DksA, observed *in vivo*. Misincorporation frequency is thought to be ∼10^−4^ (52–54), with a recent transcriptome analysis using next generation sequencing suggested ∼10^−3^ frequency of mistakes in RNA (9). However, even rarely paused complexes may cause queuing of RNAPs behind the paused RNAP (9), which would make a more difficult barrier for replication, and which appear to be detrimental to cells (9). Therefore, even a moderate increase of fidelity provided by DksA/ppGpp may lead to a considerable decrease of transcription interference with replication (also discussed below).

DksA was shown to act in the vicinity of the active centre and possibly in collaboration with the Trigger Loop (55,56), the major determinant of accuracy of NTP selection (54). Though ppGpp was recently shown to bind to ω subunit of RNAP (48–50), earlier study showed that it may also occupy a pocket in the RNAP's active centre (47). Binding of DksA and ppGpp in the vicinity of the active centre may influence the binding of non-cognate substrates or the closure of the Trigger Loop, during which most rigorous discrimination against non-cognate substrates takes place (54). Note, however, that while ppGpp potentiates the anti-readthrough activity of DksA (Figure 3C, Supplementary Figure S1C), it cannot perform this activity on its own. This observation suggests that ppGpp may influence fidelity indirectly, possibly by increasing the affinity of DksA to the elongation complex or changing the mode of their interaction. Such involvement of ppGpp may not necessarily require its binding in the active centre, but may be governed by some allosteric signals from its binding site on the ω subunit.

The only known factor that may influence fidelity of transcription in bacteria is a distant homologue of DksA, factor Gre (6–7,37), which assists RNAP in resolution of misincorporated complexes via hydrolytic activity. However, the proofreading activity may be important not for fidelity of RNA synthesis *per se*, as the translation error rate is anyway higher than the transcription error rate, but for rescuing the paused misincorporated (or generally backtracked) complexes. Indeed, the input to transcription fidelity by Gre factor of *Streptococcus pneumoniae* appeared to be even less than we observed for DksA/ppGpp in this work (9). However, deletion of Gre in this organism led to a very sick phenotype, likely due to inability to resolve misincorporated/backtracked complexes and to prevent RNAPs’ traffic jams. Consistently, Gre factors were proposed to prevent collisions of transcription elongation complexes with replication forks in *E. coli*. Our results reveal DksA/ppGpp as a new fidelity factor, which along with Gre factors, decreases formation of misincorporated complexes; DksA/ppGpp act at the stage preceding formation of a stalled misincorporated complex, while Gre acts to resolve those.

## SUPPLEMENTARY DATA

Supplementary Data are available at NAR Online.

SUPPLEMENTARY DATA
