# Supplementary material for: Bacterial global regulators DksA/ppGpp increase fidelity of transcription
Source: Nucleic Acids Res. 2015 Jan 20;43(3):1529–36. doi: 10.1093/nar/gkv003 (PMC4330370; doi:10.1093/nar/gkv003)
Supplement: SUPPLEMENTARY DATA [file supp_43_3_1529__index.html]

Bacterial global regulators DksA/ppGpp increase fidelity of transcription — SUPPLEMENTARY DATA 

# Bacterial global regulators DksA/ppGpp increase fidelity of transcription

## SUPPLEMENTARY DATA

**Files in this Data Supplement:**

- Fig S1
