# Supplementary material for: Bacterial global regulators DksA/ppGpp increase fidelity of transcription
Source: Nucleic Acids Res. 2015 Jan 20;43(3):1529–36. doi: 10.1093/nar/gkv003 (PMC4330370; doi:10.1093/nar/gkv003)
Supplement: SUPPLEMENTARY DATA [file supp_gkv003_nar-03257-x-2014-File005.docx]

**
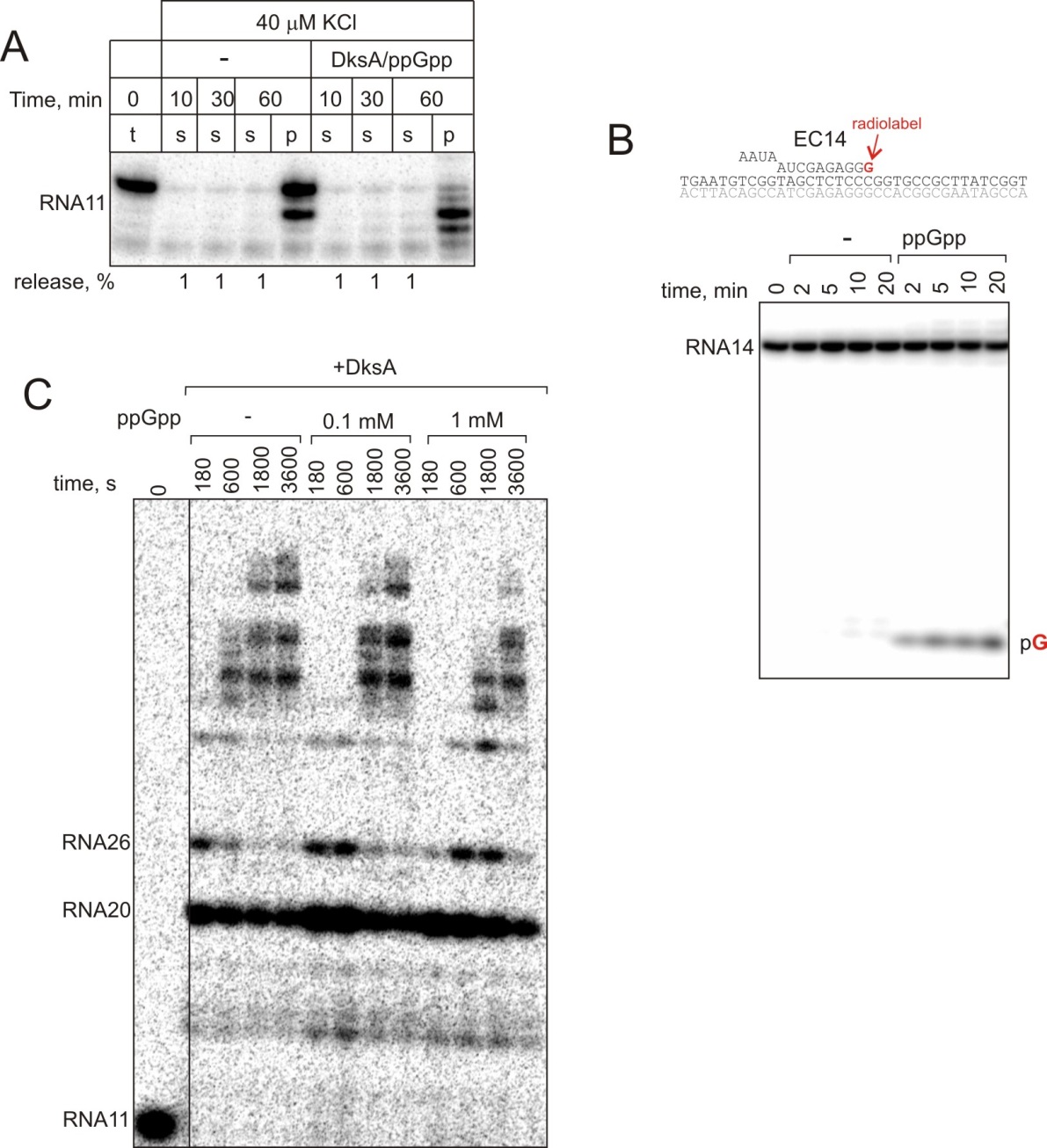
**

**Figure S1. A.** EC11 obtained from T7A1 promoter was immobilised on streptavidin beads, and supernatant (s) and/or pellet (p) fractions were analysed after various times of incubations in transcription buffer containing 100 µg/mL heparin (low salt conditions). Percentage of RNA released into the supernatant fraction is shown below the gels. Different cleavage patterns were due to ppGpp induced exonucleolytic cleavage (see panel B and text). **B.** RNA14 in assembled elongation complex (shown above the gel) was labelled at the 3’ end by incorporation of α[^32^P]-GMP. Unincorporated NTP was thoroughly removed, and complexes were incubated with 10 mM Mg^2+^ with or without 1 mM ppGpp. **C.** ppGpp potentiates anti-misincorporation activity of DksA.
